# The Efficacy and Safety of Synchronous Parallel Ultrasound for the Clinical Improvement of Cellulite Using High‐Density Handpiece

**DOI:** 10.1111/jocd.70597

**Published:** 2025-12-15

**Authors:** Michael H. Gold, Eric F. Bernstein, Anne M. Chapas, Suzanne L. Kilmer, Oleg Bedich, Ruthie Amir, David J. Goldberg

**Affiliations:** ^1^ Tennessee Clinical Research Center Nashville Tennessee USA; ^2^ Main Line Center for Laser Surgery Ardmore Pennsylvania USA; ^3^ UnionDerm New York New York USA; ^4^ Department of Dermatology Icahn School of Medicine at Mt. Sinai New York New York USA; ^5^ Laser & Skin Surgery Center of Northern California Sacramento California USA; ^6^ SofWave Medical Yokneam Illit Israel; ^7^ Cosmetic Dermatology and Clinical Research Schweiger Dermatology Group New York New York USA

**Keywords:** cellulite, skin laxity, Synchronous Ultrasound Parallel Beam Technology

## Abstract

**Background:**

Cellulite is a common aesthetic condition of the skin, predominantly affecting more than 90% of postpubertal females. Energy‐based devices (EBDs) have demonstrated efficacy in treating a variety of dermatologic concerns. A controlled thermal injury to the dermis can stimulate remodeling of the extracellular matrix (ECM), leading to skin tightening, an effect that may improve the dimpled appearance of cellulite.

**Objective:**

To assess the efficacy and safety of synchronous parallel ultrasound using a high‐density handpiece for improving cellulite appearance.

**Materials and Methods:**

Sixty subjects, aged 23–65, were enrolled in a prospective, open‐label, non‐randomized, multicenter study. Subjects underwent two treatment sessions using a novel ultrasonic system equipped with a new high‐density handpiece, applied on either one side or both sides of the thighs and/or buttocks. The follow‐up period extended up to 3 months after the second treatment. Clinical improvements were evaluated by three blinded physicians based on baseline and follow‐up photographs, using the Cellulite Severity Scale (CSS), Global Aesthetic Improvement Scale (GAIS), and Laxity Scale (LS). Satisfaction questionnaires were completed by subjects. Treatment discomfort was rated immediately after treatment using the Numerical Scale Response (NSR).

**Results:**

Sixty subjects, with a mean age of 47, were enrolled in the study. GAIS results showed improvement in 91% of the treated areas, CSS score improved by 69%, while LS score improved by 53%. Most subjects (73%) reported satisfaction. Mean pain score was 4.26 ± 2.33. No serious adverse events were reported.

**Conclusion:**

The novel ultrasound system was found to be effective and safe for improving cellulite appearance.

## 1 Introduction

Cellulite is a common aesthetic alteration affecting more than 90% of females. It is characterized by a dimpled texture of the skin surface and is associated with negative psychosocial effects [1, 2].

**FIGURE 1:**
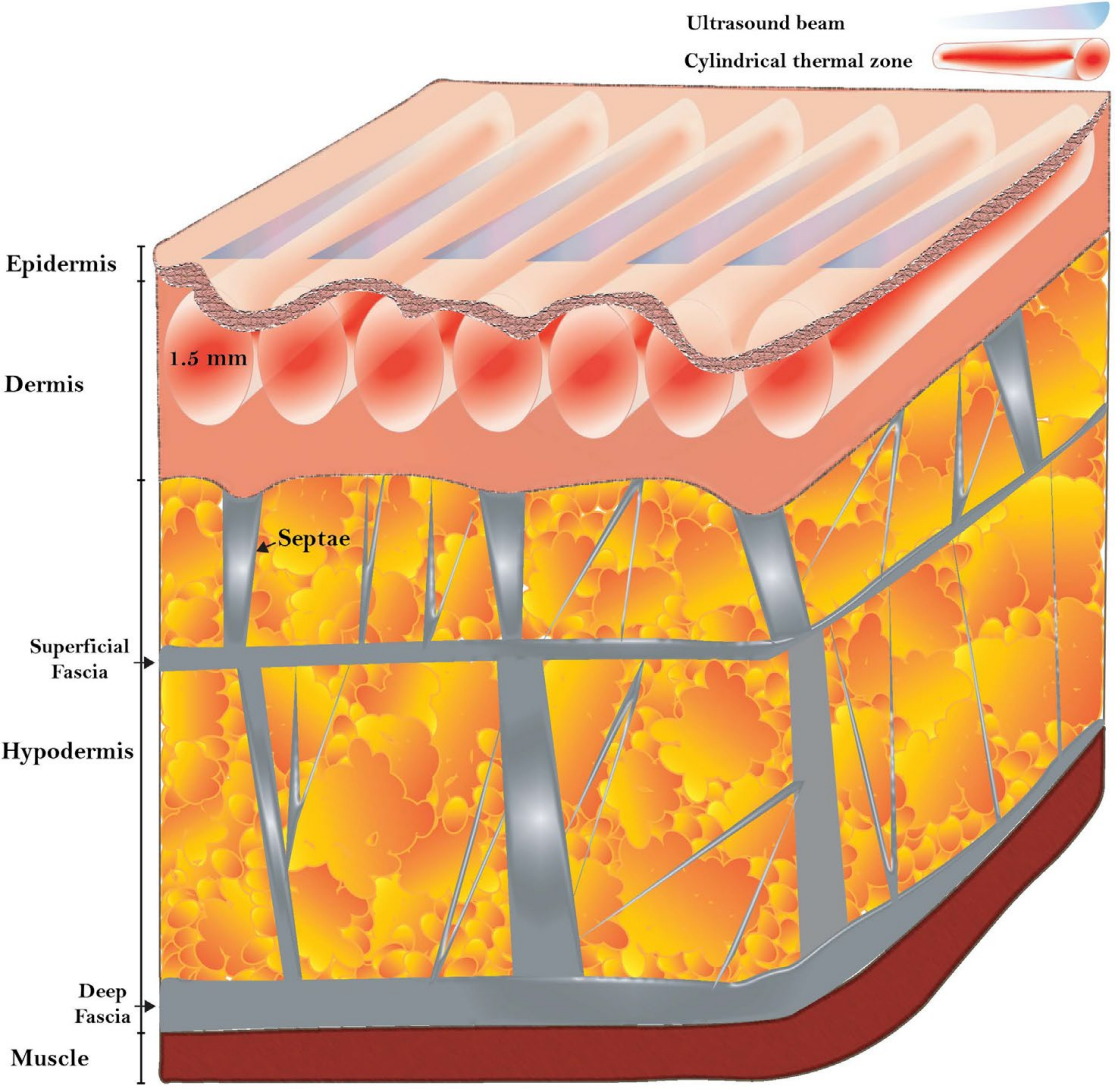
Synchronous parallel ultrasound thermal effect on skin layers. Schematic representation of the thermal effect of synchronous parallel ultrasound during its application to dimpled human skin. The high‐intensity, high‐frequency, nonfocused ultrasound beams generated by the high‐density liftHD handpiece penetrate to the dermis at depths of 0.5–2 mm, creating multiple cylindrical volumetric thermal zones. The temperature within the zone ranges from 60°C to 70°C, leading to collagen and elastin denaturation and subsequent tissue remodeling. Dermal reorganization improves cellulite appearance caused by impaired tethering of the dermis to the underlying fascia by fibrous connective tissue. (Illustration was created by SofWave Medical Ltd. using Adobe Illustrator 2025 software.)

The uneven skin appearance results from the impaired orientation of the fibrous connective tissue within the subcutaneous layer. Anatomically, the hypodermis is divided by the *fascia superficialis*, separating it into superficial and deep subcutaneous adipose tissue layers (Figure 1) [3, 4]. Within the subcutis, fibrous connective tissue septa extend from the dermis, tethering the underlying fascia and providing structural support. Alterations in the fibrous architecture, including changes in collagen content and orientation within the superficial subcutaneous tissue, can contribute to the formation of skin dimpling [2, 5].

Traditional treatments for cellulite range from invasive to noninvasive modalities, each employing distinct therapeutic strategies. Such treatments include topical agents, oral supplements, massage, fat transfer, injectables (dermal fillers and biologics), subcision, and energy‐based devices (EBDs) [2, 6, 7].

While topical agents (Methylxanthines such as Caffeine) and oral supplements aim to stimulate dermal neocollagenesis, enhance lipolysis, improve cutaneous microcirculation, and reduce lipogenesis, inflammation, and oxidative stress through a combination of various active compounds, their clinical efficacy remains limited [2, 6, 7].

Massage focused on improving the underlying impaired microcirculation and drainage deficiencies [2].

Fat transfer and injectable fillers are used to compensate for localized volume loss associated with cellulite. Beyond their volumizing effect, certain fillers such as calcium hydroxylapatite, also pose biostimulatory properties that can stimulate neocollagenesis and neoelastogenesis [2]. Subcision, by contrast, is a minimally invasive surgical technique that targets the fibrous septa within the hypodermis. By severing these tethering bands (Figure 1), subcision aims to redistribute biomechanical forces and smooth the skin surface [2].

Additional treatment options for cellulite include energy‐based therapies that utilize acoustic waves, radiofrequency (RF), laser (e.g., 810‐nm diode laser, 1064 nm Nd:YAG, 1440 nm Nd:YAG), and light‐based energies to induce controlled thermal injury in the deep cutaneous layers Both RF and light‐based technologies may be in combination with massage, vacuum etc. A series of treatments is required to obtain results. Overall, the induced damage initiates a tissue‐repair response that can lead to improvement in the appearance of cellulite [2, 6, 8].

Acoustic wave therapy enhances cutaneous microcirculation, stimulates neocollagenesis, and promotes lymphatic drainage, leading to improved skin structure and texture [2, 6].

The high‐intensity synchronous parallel ultrasound technology has shown clinical efficacy in the treatment of various dermatologic conditions, including skin laxity, wrinkles, acne scars, and cellulite [9, 10, 11, 12, 13].

Previous studies have demonstrated the safety and effectiveness of the lift applicator, which delivers seven parallel ultrasound beams to create cylindrical volumetric thermal zones within the mid‐dermis at depths ranging from 0.5 to 2 mm (Figure 1). In this prospective, open‐label, multicenter study, we evaluated the efficacy and safety of the high‐synchronous parallel ultrasound device equipped with the novel liftHD applicator, a high‐density handpiece featuring a larger transducer array for the treatment of cellulite.

## 2 Materials and Methods

### 2.1 Overview

To assess the efficacy and safety of the novel SofWave system using the liftHD applicator (SofWave Medical Ltd., Israel) for improving thigh and buttock cellulite appearance, 60 female subjects aged 23–65 years, with Fitzpatrick skin types I–IV were enrolled in a prospective, open‐label, nonrandomized, multicenter study. The clinical trial was conducted in accordance with the Declaration of Helsinki by five investigational sites across the United States of America (Main Line Center for Laser Surgery, PA; Tennessee Clinical Research Center, TN; UnionDerm, NY; Laser & Skin Surgery Center of Northern California, CA; Schweiger Dermatology, NJ). A signed informed consent and a photo consent were obtained from all subjects.

### 2.2 Inclusion and Exclusion Criteria

Inclusion criteria were healthy females, aged 18–60 years; not pregnant or lactating, and either postmenopausal, surgically sterilized, practicing abstinence, or using a medically acceptable form of birth control for at least 3 months prior to enrollment; visible cellulite in the upper thigh and/or buttock areas and actively seeking treatment; stable body weight (±5%) for at least 6 months prior to enrollment; willing to maintain body weight within ±5% throughout the study by avoiding major lifestyle changes; no history of invasive or energy‐based cellulite treatments within the 12 months prior to enrollment; no use of topical cellulite treatments within 6 months prior to enrollment, and agrees not to use any during the study except for study‐related procedures; agrees not to undergo any other cellulite treatments for 3 months following the final treatment; willing to have photographs taken of treated areas and consents to the use of de‐identified images for evaluations, publications, and presentations purposes; able and willing to comply with all scheduled visits, treatments, and study requirements; capable of understanding and signing the written informed consent.

Participants were excluded if they met any of the following: history of chronic drug or alcohol abuse; history of epileptic seizures or severe migraines; BMI ≥ 30 kg/m^2^; body weight fluctuation > ±5% within the past 6 months; use of diet pills or weight control supplements within 1 month prior to enrollment; current heavy smoker or history of heavy smoking (≥ 25 cigarettes per day) in the past 10 years; pregnant, planning pregnancy during the study, breastfeeding, or gave birth within 3 months prior to enrollment; coagulopathy or current use of anticoagulant medications; bleeding disorders or use of medications that in the investigator's opinion, may increase the risk of bruising; medical conditions that impair wound healing or immune response; active implanted electronic devices (e.g., pacemaker, defibrillator, cochlear implant, drug delivery system); known allergy to lidocaine, epinephrine, or antibiotics; active malignancy or malignancy within the past 5 years; significant medical conditions such as cardiac disease, type I or II diabetes, lupus, porphyria, or relevant neurological disorders; immunosuppressive disorders or current use of immunosuppressive medications; diagnosed hormonal imbalance (e.g., thyroid, pituitary, androgen‐related); history of lymphatic drainage disorders; dermatologic conditions (e.g., severe solar elastosis, atrophic scars, keloids, skin disorders in the treatment area); tattoos or remnants of tattoos in or near the treatment area; presence of metal or plastic implants in or near the treatment area; cognitive or language barriers preventing informed consent; ongoing psychiatric medication use; unwilling or unable to comply with study procedures or provide treatment area photographs; concurrent participation in another investigational drug or device study; any other condition that, in the investigator's professional opinion, could interfere with the study or pose a risk to the participant.

### 2.3 Treatment and Results Evaluation

Subjects underwent two treatment sessions with a high‐intensity high‐frequency, non‐focused ultrasound SofWave device using the novel liftHD applicator. Treatments were applied to one or both sides of the thighs/buttocks, targeting areas with visible cellulite. Treatment zones were cleansed, dried and marked to a standardized area of ~15 cm^2^.

Anesthetic agents included benzocaine 20%/lidocaine 10%/tetracaine 10% (BLT), lidocaine 30%, LMX 4%, tetracaine 7%, nitric oxide, and oral Toradol. Anesthesia was administered at the investigator's discretion via oral, inhalational, topical, intramuscular, or combined routes, and could include Zimmer cooling for skin surface comfort.

A thin, uniform layer of ultrasound gel was applied to the treatment area. Standardized photographs were taken before and after each treatment session, as well as at the 3‐month follow‐up visit. Immediately after each treatment, subjects were asked to rank their discomfort using an 11‐point Numerical Scale Response (NSR), where 0 indicated no pain and 10 represented the worst possible pain.

Clinical improvements were evaluated by three independent, blinded reviewers (reviewers were all experts in the field of aesthetic medicine) who assessed baseline and follow‐up images using Global Aesthetic Improvement Scale (GAIS) (−1 = worsened, 0 = no change, 1 = improved, 2 = much improved, 3 = very much improved); skin Laxity Scale (LS) (0 = absence of laxity, flaccidity, or sagging skin, 1 = slight draped appearance, 2 = moderate draped appearance, 3 = severe draped appearance); and Cellulite Severity Score (CSS) (0 = no depressions, 1 = small amount (1–4 visible depressions)/superficial‐depth depressions, 2 = moderate amount (5–9 visible depressions)/medium‐depth depressions, 3 = large amount (10 or more visible depressions)/deep‐depth depressions). The CSS was scored separately for the number of depressions and depth of depressions; the final score was the sum of both components minus 1. Baseline and follow‐up images were randomized and provided to the reviewers, who were asked to identify which image represented the baseline and which represented the follow‐up.

Subjects also completed a self‐assessment satisfaction questionnaire based on a comparison of their baseline and follow‐up images.

### 2.4 Statistical Analysis

Statistical analysis and graphical visualizations were performed using IBM SPSS version 29.0.1.0 (171), GraphPad Prism 10.4.2, and Adobe Illustrator 2025 software. Statistical tests were interpreted at a 5% significance level, with the following notation: not significant (ns) > 0.05, * ≤ 0.05, ** ≤ 0.01, *** ≤ 0.001, **** ≤ 0.0001. A 95% confidence interval (CI) was used throughout.

The blinded reviewers’ correct identification results of pre‐ and posttreatment images were assessed using a two‐sided exact binomial test, with CI calculated using the Clopper–Pearson exact method.

CSS and LS results were analyzed using a one‐sided, paired *t*‐test, while GAIS scores were evaluated using a one‐sided one‐sample *t*‐test.

Continuous variables are reported as mean ± standard deviation (SD).

## 3 Results

### 3.1 Subject Demographics and Clinical Characteristics

Sixty female subjects with a mean age of 47 ± 10 years (*R*: 23–65 years) underwent high‐intensity high‐frequency, non‐focused ultrasound treatment targeting the thighs and/or buttocks. Of these, 57 completed both treatment sessions, while three subjects discontinued after the first session, two due to noncompliance with study scheduling requirements and one who withdrew consent. An additional two subjects dropped from the study after completing both treatment sessions.

**FIGURE 2:**
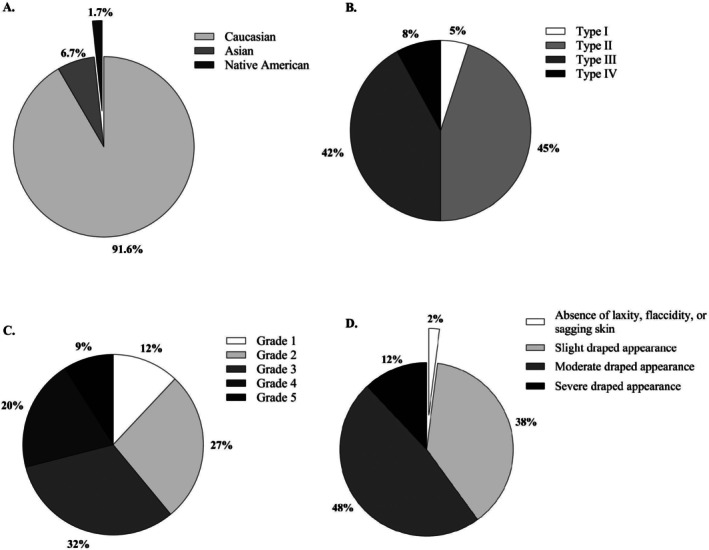
Baseline demographics and clinical characteristics of study subjects. (A) Distribution of ethnic groups. (B) Fitzpatrick skin type classification. (C) Cellulite severity based on CSS scores. (D) Skin laxity classification based on LS scores. Ethnic group and Fitzpatrick skin type data are reported for all enrolled subjects (*n* = 60). CSS and LS scores are based on baseline assessment of treated areas (*n* = 100).

The majority of subjects were Caucasian (Figure 2A), with a mean BMI of 23.42 ± 2.90 kg/m^2^ (*R*: 16.71–29.47 kg/m^2^) and Fitzpatrick skin types I–IV (Figure 2B).

A total of 100 treatment areas were included, with 40 subjects treated on both sides and 20 subjects treated on one side only. Most treated areas had a CSS grade of 3–5 (Figure 2C), and a slight to severe draped appearance according to the LS (Figure 2D). All but one subject maintained a stable body weight throughout the study; one subject experienced an 8.5% weight reduction by the 3‐month follow‐up. The mean total body weight change at 3 months compared to baseline was −0.13% ± 1.75% (*R*: −8.5%–4.0%, *p* = 0.59).

### 3.2 Treatment Parameters

Treatment areas included thighs and/or buttocks. Forty subjects (66.7%) received treatment on both the left and right sides of the thigh and/or buttock, while the remaining 20 subjects (33.3%) were treated on only one side. Overall, 18% of subjects were treated on both thighs and buttocks, 74% on the thighs only, and 8% on the buttocks only.

All treatments were performed in a continuous mode, with 3–4 passes to ensure full coverage of the treatment area. The mean number of pulses per treatment area was 165 ± 41 (*R*: 100–337), with a mean pulse energy of 4.50 ± 0.88 J (*R*: 4.60–6.60 J). A post‐cooling duration of 1 s was applied in 53% of treatments, while 2‐ and 3‐s durations were applied in 26% and 21% of treatments, respectively.

### 3.3 Efficacy Evaluation

**FIGURE 3:**
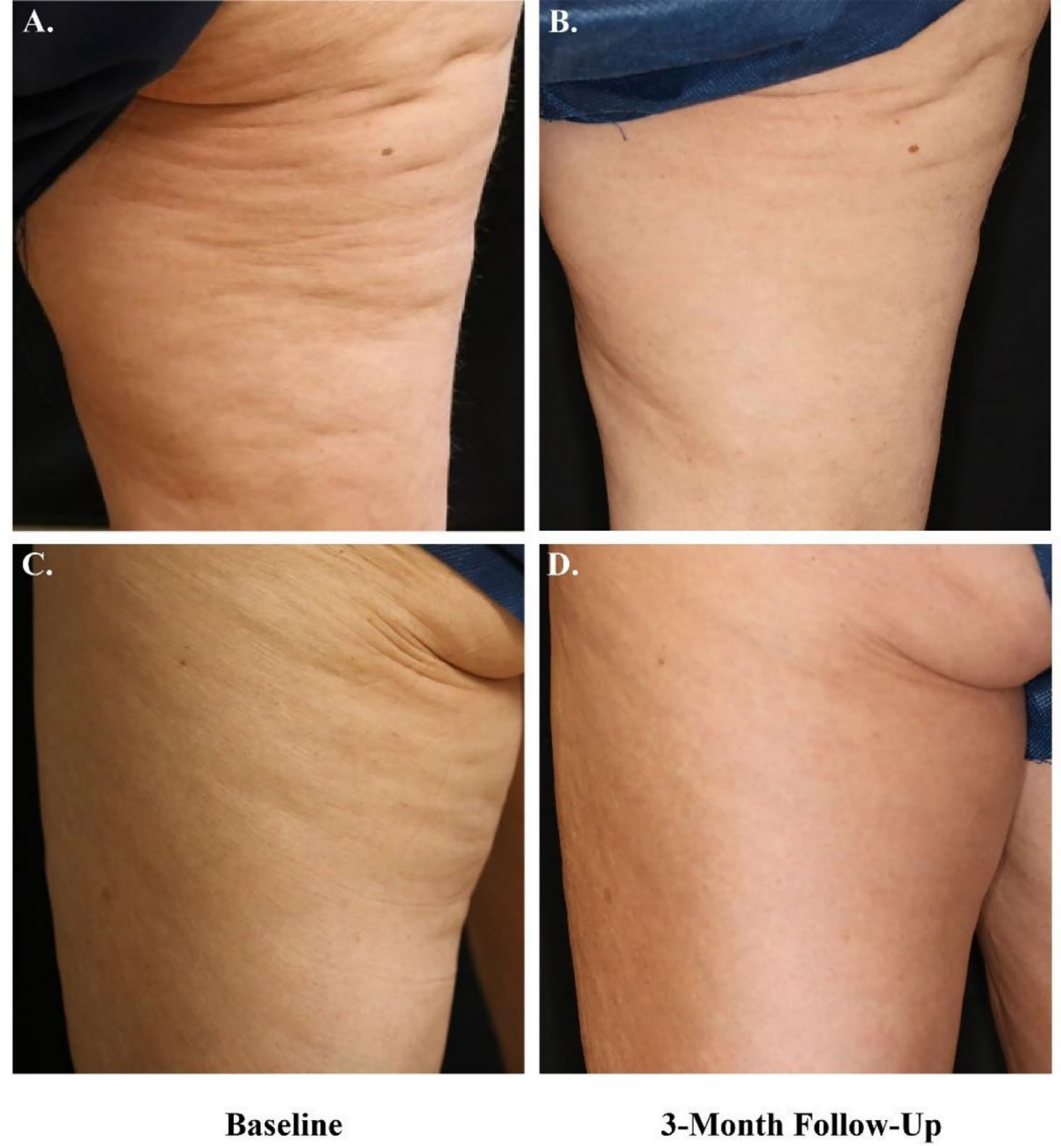
Representative pre‐ and posttreatment images of the thigh area. (A, C) Baseline. (B, D) Three‐month follow‐up. (A, B) The subject is a 65‐year‐old Caucasian female with Fitzpatrick skin type III. (C, D) The subject is a 60‐year‐old Caucasian female with Fitzpatrick skin type III.

Fifty‐five subjects completed the 3‐month follow‐up visit, contributing a total of 92 evaluable treatment areas. Two additional subjects, representing three treatment areas, discontinued the study after the second treatment session. Three independent, blinded reviewers were tasked with evaluating randomized pre‐ and posttreatment images (Figure 3), using the CSS, GAIS, and LS (Results summarized in Table 1). Prior to evaluation, reviewers were asked to correctly identify which image was baseline and which was posttreatment.

**TABLE 1:** Blinded reviewers’ assessments.

	Baseline	3‐month follow‐up	Improvement level at 3‐month follow‐up
% Correct identification per thigh^a^			92% CI [84%, 96%]
% Correct identification per subject^a^			86% CI [74%, 94%]
Mean CSS per thigh	2.58 ± 1.21	0.80 ± 1.10	69%
Mean CSS per subject	2.65 ± 1.18	1.18 ± 1.13	66%
Mean GAIS for cellulite			1.38 ± 0.86 CI [1.21, 1.56]
Mean GAIS for skin laxity			1.38 ± 0.85 [1.20, 1.55]
GAIS frequency^b^			91% improved 4% no change 5% worsened
Mean LS per thigh	1.60 ± 0.66	0.76 ± 0.59	53%
Mean LS per subject	1.66 ± 0.67	0.83 ± 0.62	50%

^a^ Correct identification of pre‐ and posttreatment images presented in a random manner.

^b^ Same results were obtained for cellulite and for skin laxity.

The correct identification rate for the 95 image pairs, defined as agreement between at least two of three reviewers was 92% (*p* < 0.0001, 95% CI [84%, 96%]).

Another assessment examined the success rate of correct identification of all treated areas per subject, thereby considering the subject as an observational unit (*n* = 57). The rate of correct identification for all treated areas within each subject was 86% (*p* < 0.0001, 95% CI [74%, 94%]).

**FIGURE 4:**
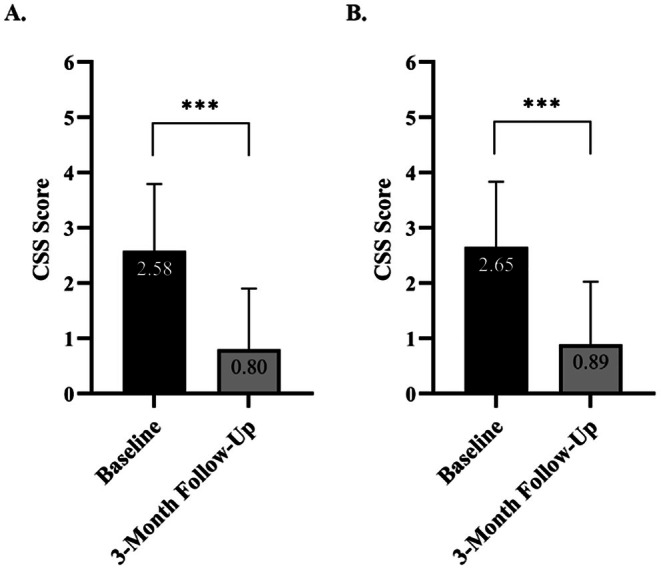
Mean CSS scores at baseline and 3‐month follow‐up. (A) Mean CSS scores based on individual treatment area assessments (*n* = 95). (B) Mean CSS scores based on subject level assessment, considering each subject as a single observational unit (*n* = 57). A statistically significant reduction in mean CSS scores was observed from baseline to follow‐up in both analyses.

Evaluation of the 95 treatment areas showed a significant 69% improvement level from the baseline CSS score, with the score decreasing from 2.58 ± 1.21 at baseline to 0.80 ± 1.10 at follow‐up (*p* < 0.001) (Figure 4A). A parallel analysis using the subject as an observational unit demonstrated a 66% improvement level, with mean scores declining from 2.65 ± 1.18 to 0.89 ± 1.13 (*p* < 0.001) (Figure 4B).

Further analysis using GAIS revealed statistically significant improvements in both cellulite and skin laxity across the 95 treated areas. The mean GAIS scores were 1.38 ± 0.86 for cellulite (*p* < 0.001, 95% CI [1.21, 1.56]) and 1.38 ± 0.85 for skin laxity (*p* < 0.001, 95% CI [1.20, 1.55]).

Based on GAIS assessments, 91% of subjects showed improvement, 4% exhibited no change, and 5% were rated as worsened by the blinded reviewers.

**FIGURE 5:**
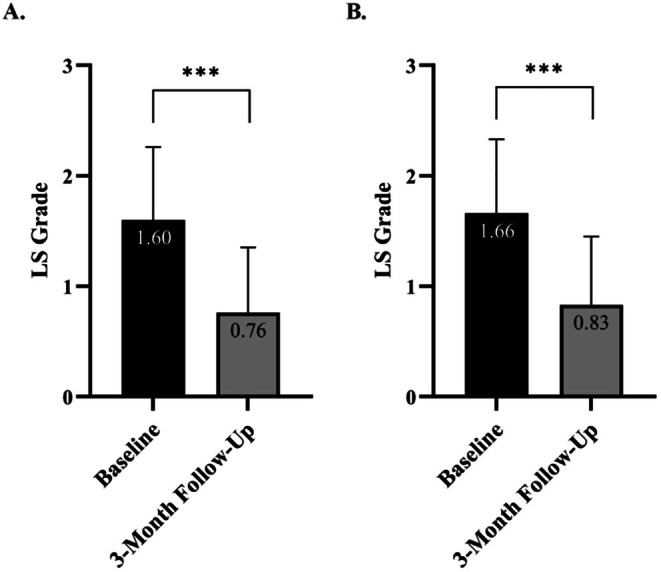
Mean LS grades at baseline and 3‐month follow‐up. (A) Mean LS grades based on individual treatment area assessments (*n* = 95). (B) Mean LS grades based on subject level assessment, considering each subject as a single observational unit (*n* = 57). A statistically significant reduction in mean LS grades was observed from baseline to follow‐up in both analyses.

LS scores for the 95 pre‐ and posttreatment areas showed a 53% improvement level from the baseline LS value, with the mean score decreasing from 1.60 ± 0.66 at baseline to 0.76 ± 0.59 at follow‐up (*p* < 0.001) (Figure 5A). A similar 50% improvement level was observed when using subjects as the observational unit (*p* < 0.001) (Figure 5B).

### 3.4 Subject Assessment

Subjects’ self‐assessment of baseline and 3‐month follow‐up images indicated high satisfaction rates, with 73% of subjects being satisfied with the results, 14% neutral, and only 13% expressing dissatisfaction with the clinical outcome.

### 3.5 Safety and Adverse Events

Topical anesthesia alone was used in 55% of treatments, while Zimmer cooling and combined pain management approaches were applied in 19% and 25% of treatments, respectively. The mean pain level reported during treatment was 4.26 ± 2.33 on an 11‐point scale, with no significant difference observed between the first and second treatments (*p* = 0.061). In most treatments, subjects reported discomfort levels ranging from 0 to 5, indicating none to mild discomfort.

Immediate posttreatment responses included erythema in 56% of treatment sessions and edema in 2%. In most cases, erythema severity ranged from minimal to moderate, with only 2% of treated areas exhibiting severe erythema. Edema was minimal in severity. All cases resolved spontaneously without intervention and were absent at all subsequent visits.

Adverse events (AEs) were reported in four subjects: One was possibly related to the device, while the remaining three were unrelated. All AEs resolved spontaneously without treatment. The device‐related AE involved a transient mild tingling sensation on the right upper posterior thigh, lasting approximately 2 h on the night following the first treatment. Notably, this sensation did not recur after the second treatment session. No unanticipated or serious AEs were reported.

## 4 Discussion

Cellulite remains a major aesthetic concern among individuals seeking cosmetic dermatologic procedures, affecting more than 90% of postpubertal females [1, 2]. In a comprehensive study by Maisel et al. [14], the characteristics and motivations of patients pursuing such procedures were examined. The most commonly reported motivations for cellulite treatment included the desire to improve skin appearance and attractiveness, and to increase self‐confidence [14].

Several treatment options are available for cellulite, including topical agents (such as methylxanthines like aminophylline, theophylline, and caffeine), oral supplements (e.g., fish oil, *Ginkgo biloba* , and Aronia juice), massage therapy, injectable treatments (such as Collagenase *Clostridium histolyticum* ), and subcision procedures (which involve inserting a needle beneath the skin to release the fibrous bands causing cellulite). Another approach involves the use of EBDs, including RF, laser therapies, and acoustic wave therapy [2, 6, 7, 8]. This study demonstrated the effectiveness of the Synchronous Ultrasound Parallel Beam (SUPERB) technology, which delivers high‐intensity high‐frequency, non‐focused parallel ultrasound beams via a new high‐density handpiece. The acoustic energy penetrates the mid‐dermis at depths ranging from 0.5 to 2 mm, elevating tissue temperature to 60°C–70°C. This temperature range is known to induce extensive collagen denaturation and protein coagulation under physiological conditions [15, 16]. The resulting coagulation zones form unique cylindrical volumetric thermal columns, with highest temperatures centered at a depth of approximately 1.5 mm.

Unlike laser and RF technologies that rely on electromagnetic radiation, ultrasound is a mechanical acoustic wave, capable of propagating deep into cutaneous layers via molecular displacement [17]. Its chromophore‐independent nature makes it safe for all skin phototypes. Additionally, the non‐focused ultrasound device used in this study provides broader dermal coverage compared to focused ultrasound technologies. A key technological innovation of this non‐focused ultrasound device is the integration of an active epidermal cooling mechanism, along with real‐time skin temperature monitoring, enabling it to induce controlled mid‐dermal injury while minimizing the risk of epidermal damage.

The localized thermal injury triggers a wound‐healing cascade initiated by ECM disruption. This response includes a brief hemostatic and inflammatory phase, followed by proliferative and remodeling phases marked by ongoing synthesis and reorganization of preexisting and newly synthesized collagen and elastic fibers [18].

Based on this scientific principle, previous studies have demonstrated the efficacy and safety of this ultrasound system for various dermatological conditions, including skin laxity, wrinkles, acne scars and cellulite, though with the use of a standard handpiece [9, 10, 11, 12, 13]. In a prior study by Wang et al. [9], the system was applied for cellulite treatment on the thighs and buttocks using the earlier lift handpiece. That study reported clinical improvements with correct identification of 89.2% of pre‐ and posttreatment images by two independent blinded physicians, a 57.4% improvement level in the mean CSS scores, 89.2% skin laxity and cellulite GAIS‐rated improvement rate, and a 43.8% improvement level in LS scores at the 3‐month follow‐up compared to baseline [9].

In the present study, the new high‐density handpiece produced comparable or slightly higher efficacy, indicating that the enhanced handpiece design may contribute to a greater clinical effect. The larger transducer array increases coverage with each pulse, reflected by a reduction in the mean pulse count per treatment—from 201 pulses with the standard handpiece to 165 pulses with the high‐density version. Additionally, the proportion of treatments applying three passes increased from 26% to 44%, while the percentage of treatments applying four passes decreased from 74% to 56% with the high‐density handpiece. Shortening treatment durations for large areas such as the thighs and buttocks may offer significant benefits for both clinicians and patients. Importantly, as in the Wang et al. [9] study, no cases of significant pain, tenderness, skin dyspigmentation, erosion, ulceration, necrosis, or serious AEs were reported, further supporting the device's high safety and tolerability.

Objective clinical evaluations were further supported by participant‐reported outcomes, with 73% of subjects expressing satisfaction with the results. The alignment between expert evaluation and patient satisfaction underscores the visible and significant nature of the clinical outcomes (Figure 3). This observation is particularly important given the frequent misalignment between patient expectations and clinical results in aesthetic procedures, where subjective perception often diverges from objective assessments.

To address the limitations of this study, future investigations should include extended follow‐up periods of 12–18 months to assess the durability of results, as dermal remodeling may continue over several months or even years [19]. Histological analysis of pre‐ and posttreatment skin specimens could elucidate the molecular mechanisms underlying clinical improvements. Additionally, the inclusion of a control arm and the integration of quantitative imaging tools would allow for more objective, standardized outcome assessment.

We also propose exploring combination therapies involving this ultrasound technology with topical agents or biostimulatory injectables to further enhance dermal neocollagenesis, neoelastogenesis, lipolysis, microcirculation, while reducing lipogenesis, inflammation, and oxidative stress—mechanisms relevant to the pathophysiology of cellulite. This approach may be particularly effective given that cellulite results from a multifactorial process involving skin laxity, formation of fibrous bands, and fat pad herniation [9].

In summary, this prospective, open‐label study demonstrates that high‐intensity, synchronous parallel ultrasound delivered through a novel high‐density handpiece significantly improves the appearance of cellulite on the thighs and buttocks. The treatment was safe, well‐tolerated, and produced consistent clinical improvements supported by both expert evaluation and subject‐reported outcomes.

## Conflicts of Interest

**Michael H. Gold** is a consultant and performs clinical research for SofWave. **Eric F. Bernstein has** Equity SofWave. **Anne M. Chapas is** a paid investigator for the study, and study equipment was provided by Sofwave. **Suzanne L. Kilmer is on the board of Directors for Sofwave** and perform clinical research for SofWave. **Oleg Bedich** was an employee of Sofwave. **Ruthie Amir is** an employee of SofWave Medical in charge of clinical and regulatory. **David J. Goldberg:** The study was funded, in part, with a research grant to Schweiger Dermatology Group.

## Data Availability

The data that support the findings of this study are available from the corresponding author upon reasonable request.
